# Neuroendocrine carcinoma of the ovotestis: A case report and review of literatures

**Published:** 2016-12

**Authors:** Tahereh Ashrafganjoei, Ainaz Sourati, Mahdiss Mohamadianamiri

**Affiliations:** 1 *Department of Obstetrics and Gynecology, Preventative Gynecology Research Center (PGRC), Imam Hossein Hospital, Shahid Beheshti University of Medical Sciences, Tehran, Iran.*; 2 *Department of Radiotherapy and Oncology, Shahid Beheshti University of Medical Sciences, Tehran, Iran. *; 3 *Department of Obstetrics and Gynecology, Firoozgar Hospital, Iran University of Medical Sciences, Tehran, Iran.*

**Keywords:** *Neuroendocrine carcinoma*, *Ovary*, *Carcinoid tumors*

## Abstract

**Background:**

Neuroendocine carcinoma of the gynecologic tract is rare and poses a significant clinical challenge because of tumor heterogeneity and lack of standardized guidelines for treatment. Ovotestis refers to the histology of a gonad that contains both ovarian follicles and testicular tubular elements. Ovotesticular disorder of sexual development occurs in fewer than 10% of all disorders of sexual development. Gonadal tumors with malignant potential occur in 2.6% of all cases of ovotesticular disorder of sexual development.

**Case:**

Here we represent a 77-year-old woman with primary amenorrhea, infertility and 10cm solid mass in left adnex with 46 XY in karyotype with ovotestis neuroendocrine neoplasm in pathology report which was treated with a multi-modality manner including surgery and chemotherapy but she came back with pulmonary metastasis after 2 cycles of chemotherapy. For women who present with a stage 1 primary ovarian neuroendocrine tumor the prognosis is excellent with greater than 90% survival. Neuroendocrine tumor of the ovary represents 3 % of all neuroendocrine tumors. The prevalence of ovotestis is 1/20000 births. For women with more advanced disease, the prognosis is poor. Neuroendocrine carcinoma of the ovary is a rare and aggressive tumor commonly associated with other surface epithelial and germ cell neoplasms. The prevalence of ovotestis is 1/20000 births and gonadal malignancies are the most reported neoplasm affected the ovotestis. Here we report a case of ovotestis which is presented with neuroendocrine carcinoma and poor prognosis.

**Conclusion:**

Neuroendocrine carcinoma of the ovary is a rare and aggressive tumor commonly associated with other surface epithelial and germ cell neoplasms. The prevalence of ovotestis is rare and gonadal malignancies are the most reported neoplasm affected the ovotestis.

## Introduction

Neuroendocrine carcinomas (NECS) are originated from endocrine cells of the diffuse neuroendocrine system, which are mainly from gastrointestinal tract, lungs and pancreas (1). Neuroendocrine neoplasms of the testis are unusual tumors and represent more than 1% of all testicular tumors (2). They are rarely seen in the female genital system, and most commonly in the ovaries and cervix. NEC occurs very exceptional in another part of the genital trace (e.g, the endometrium or vagina) (3). An analysis of the SEER database shows that the incidence of neuroendocrine tumor is increasing (1).

Tumor differentiation and tumor grade often are correlated with mitotic count and Ki-67 proliferation index (4). In most cases, well-differentiated, low grade tumors have a mitotic count of less than 2/10 high-power field (HPF) and Ki67 index of less than 3%. Well differentiated intermediate-grade tumors usually have a mitotic count of 2 to 20/10 HPF and/or a Ki-67 index of 3-20%. In high-grade tumors, the mitotic count usually exceeds 20/10 HPF and/or the Ki-67 exceeds 20% (4). In addition the margin status (Negative or positive), the presence of vascular or perineural invasion should be included in the pathology report, which has been suggested that these factors may also have prognostic significance (5).

In the recent decade, there has been an increased incidence of neuroendocrine tumors: which may reflect improvements in standardized classification criteria, and diagnostic recognition (1). Genital neuroendocrine tumors are rare, with limited prospective data to guide management. Resection is the primary treatment approach for localized low-grade neuroendocrine tumors with close follow-up. Whether patients with intermediate grade tumors should receive additional therapy remains a choice based on the individual clinical analysis (6).

Ovotesticular is characterized by the presence of ovarian and testicular tissues in the same individual. Most cases have a 46,XX karyotype, while 7% have 46,XY karyotype and 10-40% exhibit chromosomal mosaicism. Many patients with ovotesticular disorder have a uterus. Müllerian duct structures typically have developed on the gonad side(s) not with no testicular tissue. The gonads may be ovotestis, or they may be a combination of an ovary on one side and a testis or ovotestis on the other side (7).

Here we present a case of neuroendocrine tumor of ovotestis and its presentation, pathologic and genetic findings, and management. 

## Case report

A 77-year-old woman with a history of primary amenorrhea and infertility was admitted for pelvic pain. In pelvic exam, the patient had was a phenotypically developed female with normal external male genitalia and there was fullness in left adnex. Abdomino pelvic Computed Tomography (CT) scan and sonography showed a 10 cm solid mass in left adnex without any metastasis. Complete blood count and CA 125 tumor marker were analyzed. All laboratory tests showed normal range. 

**Figure 1:**
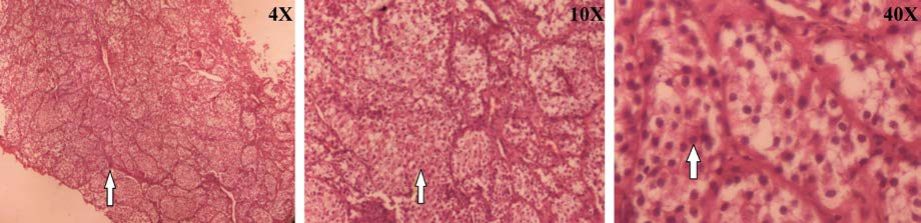
Neuroendocrine neoplasm: small nests in rich vascular background. Cells have finely granular amphophilic cytoplasm

During the laparotomy we observed a left adnex mass of 7×10 cm in diameter and an atrophic uterus with a 1.5 cm myoma without cervix separate from the left adnex and a small mass in right adnex. A left oophorectomy was performed and sent for frozen section and pathology review after that hystrectomy and right oophorectomy was performed. Histopathologic examination revealed bellow: pelvic mass resection, frozen and final diagnosis (Figure 1):

- Neuroendocrine tumor
- Tumor size: 8.75 cm
- Tumor is limited to capsule
- No vascular or prineural invasion is seen.
- Mitosis count 12 per 10 HPF (intermediate-grade tumors).
- Ki 67: Nuclear positivity in 30% of tumor cells and remnant of uterus, immature testis, ovotestis with immature seminiferous tubules. Intra abdominal fluid cytology and omental tissue was negative for malignancy.
- karyotype was 46XY which verified the ovotestis.

Adjuvant chemotherapy with 6 cycles Etoposide + cisplatine were then administered but pulmonary metastasis has been happened after two course of chemotherapy. Based on medical Ethics Committee of Shahid Beheshti University of Medical Sciences, a written informed consent was obtained from the patient for publication of this article and accompanying images. 

## Discussion

Lee *et al* presented a 40-year-old woman with a 30 cm pelvic mass. After the operation, she was diagnosed of stageIa NEC of left ovary. The patient received six cycles of paclitaxel-carboplatin chemotherapy postoperative similar to this case report primary neuroendocrine carcinoma presented with an adnexal mass (8). Berdjis and Mostofiin presented a series of 10 cases of carcinoid tumors of the testis: 2 of the patients died of 4-6 years (9).

Zavala-Pompa *et al* described 3 cases described can be categorized as low-grade (well-differentiated) tumors, while 1 case likely corresponds to an intermediate-grade (moderately differentiated) tumors like the case which was mentioned in this report (10). Crochet *et al* reported a primary neuroendocrine carcinoma of fallopian tube which presented with an adenexal mass. They performed laparotomy and staging and adjuvant chemotherapy 6 cycles of carbopatin/ etoposide. She was alive 9 months after the diagnosis with no evidence of relapse (11). 

Like in this case report. primary neuroendocrine carcinoma presented with an adnexal mass, but pulmonary metastasis has been happened during chemotherapy and the origin of tumor was ovotestis. Gonadal Malignancies are the most reported neoplasm affected the ovotestis. Here in our case ovotestis is presented with neuroendocrine carcinoma. 

## Conclusion

Primary carcinoid tumors of the ovary account for less than 5% of all carcinoid tumors and for less than 0.12% of all ovarian neoplasms. Neuroendocrine tumor of the ovary represents 3% of all neuroendocrine tumors. The prevalence of ovotestis is 1/20000 births. For those patients in whom the diagnosis is known. Hysterectomy for early stage disease and adjuvant chemotherapy with etoposide/ cisplatin even without evidence of nodal metastasis should be considered as part of a multi-modality therapeutic strategy (3).

Neuroendocrine carcinoma of the ovary is a rare and aggressive tumor commonly associated with other surface epithelial and germ cell neoplasms. The prevalence of ovotestis is rare but we should consider it in patients with adnexal mass and in patients with simptoms like amenorrhea we should consider gental system anomalies to diagnosis of rare ovarian cancers in early stage. 
